# Analysis of Location Selection of Public Service Facilities Based on Urban Land Accessibility

**DOI:** 10.3390/ijerph18020516

**Published:** 2021-01-10

**Authors:** Wei Wang, Zihao Zhou, Jun Chen, Wen Cheng, Jian Chen

**Affiliations:** 1 School of Transportation, Southeast University, Nanjing 210096, China; wwang@seu.edu.cn (W.W.); chenjun@seu.edu.cn (J.C.); 220183102@seu.edu.cn (J.C.); 2 Key Laboratory of Cold Region Urban and Rural Human Settlement Environment Science and Technology, School of Architecture, Harbin Institute of Technology, Harbin 150001, China; hit_chengwen@hit.edu.cn

**Keywords:** location of public service facilities, urban land accessibility, correlation analysis, carbon emission

## Abstract

Urbanization has been a flourishing process in a wide range of developing countries. The planning and construction of public service facilities is a crucial component of this process. Existing planning methods of public service facilities focused on macroscopic indicators like population and GDP. In this way, accessibility and transportation conditions were neglected. Four typical counties in China were selected as samples where travel surveys and questionnaire surveys on public service facilities were conducted. Taking education and medical care as representative public service facilities, this study used geographic information processing to connect the locations of public service facilities at all levels with the urban land accessibility. Then, analysis of variance was used to obtain correlations between the level of public service facilities and the urban land accessibility. The results showed that the urban land accessibility of locations of public service facilities follows a normal distribution. Categories of facilities showed significant difference on urban land accessibility. Therefore, intervals of urban land accessibility of locations of public service facilities within one standard deviation from the mean were constructed by category. These intervals built a connection between transportation conditions with locations of public service facilities. Corresponding relation of carbon emission of facility-related trips and urban land accessibility was established as an example of an application. Carbon emissions caused by facility-related trips can be reduced by locating facilities at locations with appropriate urban land accessibility.

## 1. Introduction

Urbanization has been a flourishing process in a wide range of developing countries. Take China as an example; at the end of 2016, the national urban built-up area was 54,000 square kilometers. The national urban built-up area has increased by 47,000 square kilometers, or 6.7 times greater since the end of 1981 [1]. In 2014, the central government proposed a new urbanization strategy. The core of the new urbanization strategy is to control the scale of urban land use. China’s urban development has changed from incremental expansion in the past to content-based growth.

With demand increasing, urban planning in large cities has become an increasingly difficult problem to solve. Therefore, county-level towns are increasingly choosing to urbanize at the original location to ease the pressure on large cities and promote their own development. In the process of urbanization at the original location, the construction of urban public service facilities is key. However, existing urban master planning can only plan for service facilities based on indicators such as population and GDP. There is currently no guidance for specific locations, nor is there consideration of the relationship between the locations of public service facilities and the transportation conditions of the location. Existing planning methods could make public services too distant for some residents. Ignorance on transportation conditions can impede the optimization of the structure of transport mode. It can be said that the relatively slow development of county-level towns in China is largely attributable to the unreasonable allocation and location of public service facilities in those towns.

## 2. Literature Review

Since the development of facility location theory in the 1960s, a relatively complete theoretical system has been formed. Among existing theories, discrete location theory is the most widely used. The basic problems included in the theory are the location set covering problem, maximum location covering problem, P-center problem, P-median problem, and flow-capturing location problem (FCLP) problem.

Toregas et al. [2] proposed the location set covering problem in 1971 to deal with the location of emergency facilities and considered how to minimize the number of facilities based on covering the needs of all demand points. However, the location set covering problem does not consider actual demand differences between demand points. Church and ReVelle [3] proposed the maximum location covering problem in 1974, aiming to determine the maximum demand point within the covered area. Meanwhile, Hakimi [4] proposed the P-center problem in 1964. He considered how to select P facilities while ensuring that all hotels are covered to minimize the distance of the demand point that is the farthest from the facility. Hakimi [5] then proposed the P-median problem and studied how to minimize the product of the distance from the demand point to the facility and the actual demand. Later, the FCLP problem was developed to solve the location problem considering construction costs. Existing research on location has thus focused on exploring the relative positional relationship between demand and supply, as well as supply and supply. However, less attention has been paid to the effect of actual transportation conditions on location. This study aimed to help fill this gap.

Besides mathematical theory of location selection of public service facilities, recent research tends to apply more complicated algorithms to make the computing environment closer to reality. Zheng et al. [6] developed a geographic information system (GIS)-based hybrid model combining the widely used analytic hierarchy process (AHP) multi-criteria analysis method with the Huff model that predicts the number of visiting customers to determine the optimal facility for collaboration and service as a parcel-pickup point. Song et al. [7] utilized the new gravity P-median model to conduct an empirical study for the spatial equilibrium layout of general hospitals in the urban area of Nanjing City. Cheng et al. [8] implemented a modified immune algorithm (MIA) to find the optimal solutions for placing residential care facilities.

As Ni et al. [9] stressed, the spatial distribution of urban service facilities is largely constrained by the road network. Even if complicated algorithms may be more accurate, the ignorance of actual road networks and transit services makes it too idealized. Our research sets the purpose to find a balance between macroscopic and microscopic scale, and we believe urban land accessibility is a good point to start.

Since the 1990s, the rapid development of geographic information systems has helped facilitate research on the planning of public service facilities [10]. Among the research objects, medical facilities, educational facilities, and parks and green spaces have attracted the most attention.

Like traditional facility location theory saw itself as exploring the relative positional relationship between demand and supply, or supply and supply, accessibility can also be seen as the combination of supply and demand [11]. Kong et al. [12] classified public facilities and explored the difference in facilities based on spatial interaction.

Researchers in this area have also provided an abundance of accessibility measurement methods. Common ones include the container method, shortest distance method, least travel cost method, cumulative opportunity method, kernel density method, two-step floating catchment area method, and gravity model method [13,14,15]. However, these measurement methods are mainly based on the accessibility assessment of locations and are influenced by the temporal geography school. Research on individual spatiotemporal accessibility, which is more in line with social reality, has received increasing attention. This approach focuses on the effects of real-time traffic factors (e.g., the opening hours of public service facilities, choice of individual transportation mode, departure time) on the accessibility and fairness of public service facilities [16,17]. Ahmed et al. [18] supposed accessibility is a time-varying variable that changes according to traffic condition. Previous studies have used accessibility as an indicator to evaluate public service facility planning, reflecting the importance of accessibility for efficient facility operation. While we can infer that using urban land accessibility as a basis for facility location has high theoretical and practical value, there is little research in this area.

## 3. Method for Analyzing the Correlation between Public Service Facility Category and the Transportation Conditions of Locations

### 3.1. Classification of Public Service Facilities

Detailed classifications can be found in the Code for Urban Public Facilities Planning by China’s Ministry of Housing and Urban–Rural Development. Educational facilities include primary and secondary schools, special-education schools, secondary vocational schools, and higher education institutions. Table 1 shows the specific categories.

Meanwhile, medical facilities include hospitals, primary medical and health facilities, and professional public health facilities, which are classified as shown in Table 2.

Among these classifications, since primary schools, secondary schools, and preschools typically involve more commuting, urban land accessibility has a greater effect on their service capabilities. As such, they were the main research focus of this study. Secondary vocational schools, colleges, and universities usually involve less commuting and can therefore be combined into one major category for statistical analysis.

Regarding existing facility classification standards, this study mainly investigated the correlation between the level of public service facilities in county-level towns and the transportation conditions of the facilities, as well as the correlation between the county where the public service facilities are located and the transportation conditions of the facilities.

### 3.2. Urban Land Accessibility Calculation Method

**Figure 1:**
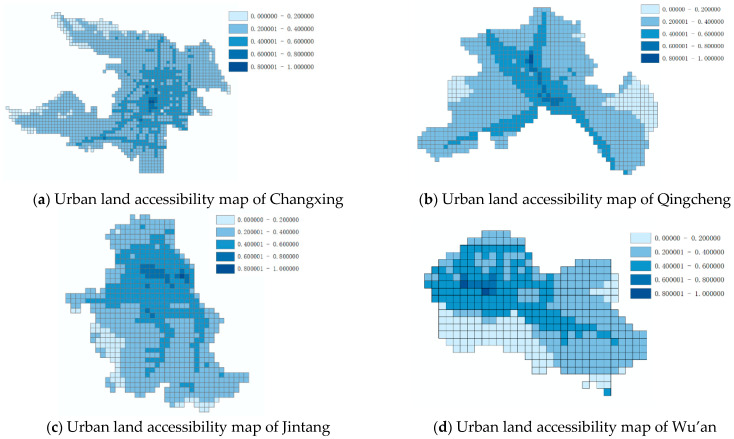
Urban land accessibility map.

We selected an appropriate length to rasterize urban land, calculate the accessibility of a certain grid of urban land *i* to all other grids according to the travel time between the grid cells and the grid cell importance coefficient, and add the external accessibility values of grid *i* to all other grids to obtain the external accessibility of grid *i*. This is divided by the maximum external accessibility value of the grid to obtain the relative external accessibility of grid *i* in the range of 0–1. Internal accessibility is usually measured by road network density, which also needs to be processed into relative values. Finally, we comprehensively considered internal and external accessibility to obtain the comprehensive accessibility of a grid. This study considered four typical counties in China as data samples and used 400 m × 400 m as the grid cell size to calculate accessibility. The calculation of urban land accessibility is shown as maps in Figure 1.

### 3.3. Spatial Connection between Facilities and Comprehensive Accessibility

Using the spatial connection function in ArcGIS, we can link the attributes of two elements in the same location in space. This study used the spatial connection function of ArcGIS to connect public service facilities and the locations of the facilities. The category number and urban land accessibility were assigned to the public service facilities as two attributes to complete the one-to-one correspondence between the classification of public service facilities and their transportation conditions.

The appropriate classification was selected to assign the category number to the point elements of the public service facilities, and ArcGIS was used to summarize the statistical attributes of each category. Then, the attribute table of the point elements was imported into SPSS (IBM, Chicago, the United States) for variance analysis to study whether a correlation existed between the category of public service facilities and the transportation conditions of the locations of the facilities.

To ensure representative counties, typical counties were selected from each of China’s climate zones to analyze separately the correlation between public service facilities at all levels and the transportation conditions of the locations of the facilities.

### 3.4. Variance Analysis

The public service facility category and the town where a facility is located are categorical variables, while the transportation conditions of the locations are numerical variables. Variance analysis was thus used to analyze their correlations. This study first considered separately the effect of the town and level of public service facility on the transportation conditions of the locations of the facilities. Then, interactive two-way analysis of variance was conducted.

The null hypothesis and alternative hypothesis of the analysis of variance are

(1) $$H_{0}:\;\mu_{1}=\mu_{2}=\ldots =\mu_{k}$$

$H_{1}$: K population means are different or not exactly the same.

Application conditions:

(1) Independence; that is, the observation object is an independent random sample at each level of the research factor.
(2) Normality; that is, the dependent variable at each level should obey normal distribution.
(3) As for the homogeneity of variance, the variance of the maximum/minimum value is generally considered to be less than 3, which means the analysis result is stable.

The four typical counties we selected can be approximated as an independent random sampling of each climatic zone. Surveying all public service facilities in all towns in these counties is equivalent to surveying the whole population, which also satisfies independence.

SPSS was used to perform the K–S normality test and generate a Q–Q plot (quantile plot) to determine whether the dependent variables at each level obey normal distribution. The null hypothesis of the K–S test is that the population satisfies normal distribution. Thus, when significance > 0.05, the null hypothesis cannot be rejected; that is, the population satisfies normal distribution. The Q–Q plot is a quantile plot. If the scatter points are roughly on the oblique line from the lower left corner to the upper right corner, the population is considered to satisfy normal distribution [19,20]. The variance of the dependent variables at each level was calculated to check whether the variance was homogeneous.

After the data were verified to meet the three application conditions, the transportation conditions of the locations of the public service facilities were used as dependent variables, and the level of public service facilities and the county-level towns where they are located were used as independent variables. The level of public service facilities is an ordinal categorical variable that can be assigned as a natural number using one-way analysis of variance. Climate zone, however, is a nominal categorical variable. Thus, the univariate linear model of the general linear model in SPSS was used to analyze whether its effect on the transportation conditions of the public service facilities was significant.

## 4. Case Study of the Correlation between Educational Facility Categories and the Urban Land Accessibility

### 4.1. Analysis of the Urban Land Accessibility of the Locations of Education Facilities of Typical Counties

For the research object, this study selected typical counties distributed in four typical climate zones across China: Jintang County, Sichuan Province, in the southwest mountainous area; Qingcheng County, Gansu Province, in the Loess Plateau; Changxing County, Zhejiang Province, in the eastern coastal region; and the county-level city of Wu’an in Hebei Province in the North China Plain.

Travel and questionnaire surveys were carried out in four typical counties in China in October 2018. 3600 facility-related travel data, 10,800 stated preference questionnaire of public service facility data were collected by investigating 3600 households.

Questionnaire data included ID, type of destination, travel frequency, traffic mode and trip distance. Trip data included ID, longitude and latitude of OD(origin and destination), traffic mode and specific name of OD.

Based on Code for Urban Public Facilities Planning by China’s Ministry of Housing and Urban–Rural Development and considering the actual situation, educational facilities were classified into six categories, which are preschool, elementary school, junior high school, high school, secondary vocational school, and higher education, with analysis made on the correlation of their classification and climate zones with the urban land accessibility. Since there are relatively few special-education schools, and they are not statistically significant, they were not analyzed.

Using the spatial connection function in ArcGIS, the comprehensive urban land accessibility was assigned as an attribute to point elements representing various levels of educational facilities. These were classified and summarized after being exported to Excel (Table 3).

**Figure 2:**
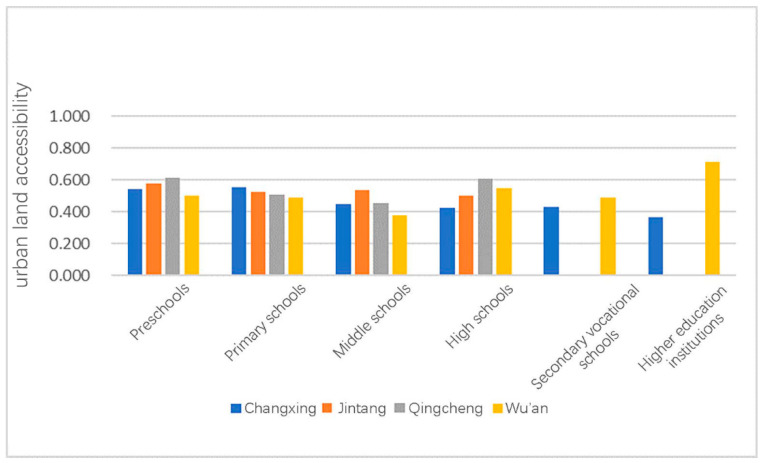
Graph of the average comprehensive urban land accessibility of locations of educational facilities at all levels in a typical county.

The averages of the four towns were then compared (Figure 2).

Except for the small sample size of colleges and universities, which caused a relatively large data fluctuation, other educational facilities at all levels had small data fluctuations between different towns, and the difference between the maximum and minimum value did not exceed 0.2. Thus, it was preliminarily determined that the average urban land accessibility of locations of educational facilities at all levels had nothing to do with the climate zone. The four counties were then combined and analyzed to obtain a more stable and accurate value.

### 4.2. Analysis of the Correlation between Educational Facility Categories and the Urban Land Accessibility

#### 4.2.1. Hypothesis

Based on the preliminary processing and data visualization in the previous section, we could preliminarily conclude that the urban land accessibility of locations of educational facilities at all levels had nothing to do with the climate zone. Thus, the null hypothesis is climate zone or category of facilities cannot significantly affect urban land accessibility. The alternative hypothesis is climate zone or category of facilities does have significant effect on urban land accessibility. They are created as:

(2) $$H_{0}:\;\mu_{1}=\mu_{2}=\ldots =\mu_{k}$$

$H_{1}$: K population means are not exactly the same.

#### 4.2.2. Verify Application Conditions

(1) Independence test

This study selected typical counties across the country, which can be approximated as independent random sampling.

(2) Normality test

The normality test should separately test the normality of the population sample and the classification sample. SPSS was used to perform a normality test and draw a Q–Q plot (a quantile plot). If most of the scatter points of the generated Q–Q plot are located on the diagonal line, it means the sample demonstrates a normal distribution.

In the Table, 1 represents preschool; 2 represents primary school; 3 represents middle school; 4 represents high school.

**Figure 3:**
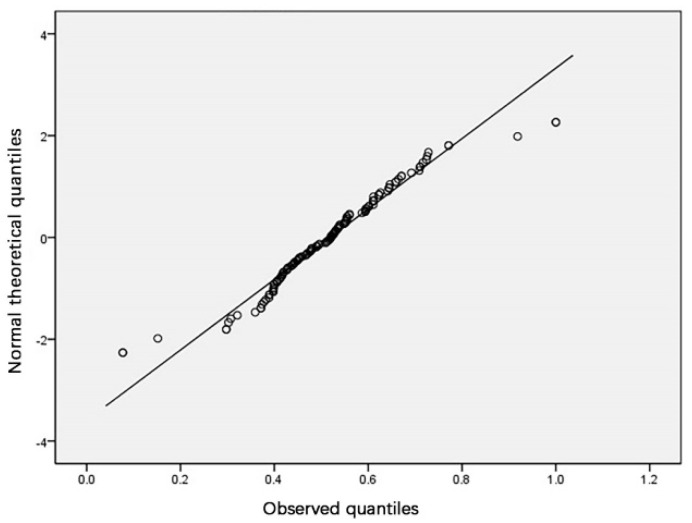
A standard Q–Q plot of the overall urban land accessibility of the sample.

**Figure 4:**
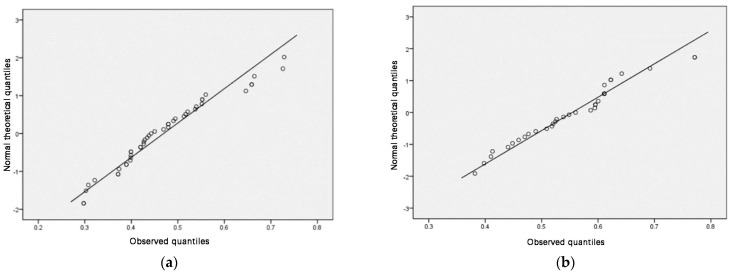

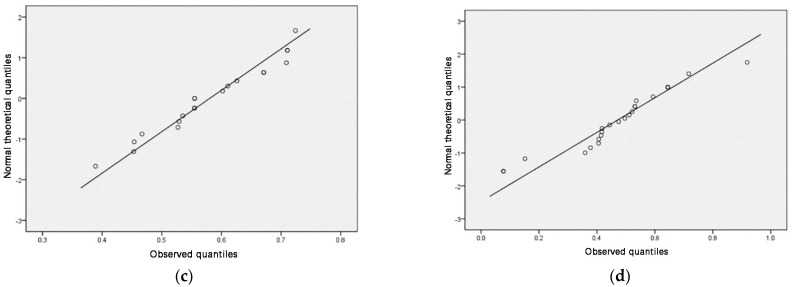
(**a**) Q–Q plot with the town as a factor (Changxing City), (**b**) Q–Q plot with the town as a factor (Jintang County), (**c**) Q–Q plot with the town as a factor (Qingcheng), (**d**) Q–Q plot with the town as a factor (Wu’an City).

**Figure 5:**
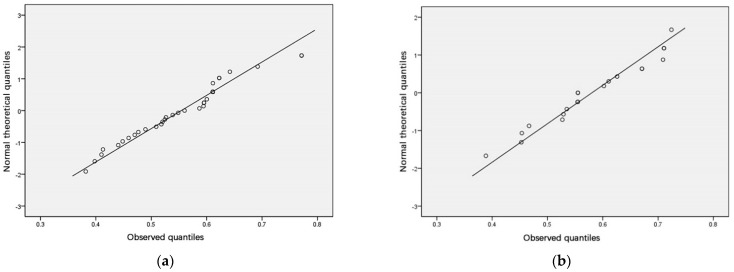

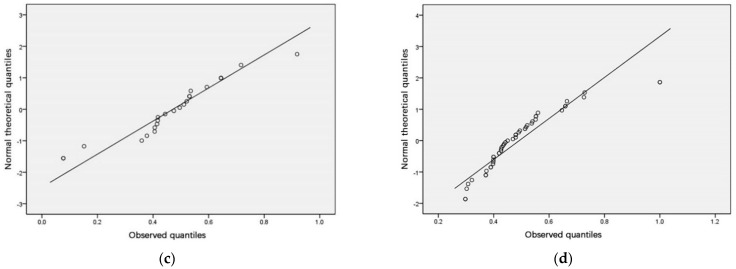
(**a**) Q–Q plot with the category as a factor (kindergarten), (**b**) Q–Q plot with category as a factor (primary school), (**c**) Q–Q plot with category as a factor (junior high school), (**d**) Q–Q plot with category as a factor (high school).

According to the above analysis results, the sample population (Table 4, Figure 3) and the classification by region (Table 5, Figure 4) were in accordance with normal distribution, and the fourth type of classification by education facility category (Table 6, Figure 5) was slightly skewed and did not meet normal distribution. Considering that other categories and the population both met normal distribution, slight skewness can be approximated as normal distribution.

(3) Homogeneity of variance

Means were calculated and compared in SPSS. Table 7 and Table 8 show that the standard deviations are not different for either type of classification method and the maximum value/minimum value was less than 3, which meets the requirement of homogeneity of variance.

#### 4.2.3. Variance Analysis

Variance analysis was performed in SPSS. The comprehensive urban land accessibility was used as the dependent variable, and the educational facility category was used as a factor to analyze whether the category would have a significant effect on the comprehensive urban land accessibility. Table 9 shows that the category of educational facilities had a significant effect on the comprehensive urban land accessibility (*p* < 0.05).

Because the number of junior high schools, secondary vocational schools, and universities is relatively small, junior high schools were merged into high schools, and the classifications of kindergartens, elementary schools, and middle and high schools were imported into SPSS. In each category, the comprehensive urban land accessibility was used as the dependent variable, and the county-level town was used as a factor to perform analysis of variance separately to study whether there were differences in the accessibility of educational facilities in different county-level towns.

Table 10, Table 11 and Table 12 show that, under each category, the significance of county-level towns for the comprehensive urban land accessibility was greater than 0.05. The null hypothesis cannot be rejected; that is, different towns will not cause the accessibility of the facilities of kindergartens, elementary schools, and middle and high schools to be significantly different, and different towns do not affect the urban land accessibility.

The category and county-level towns were used together as factors to study their interaction. Table 13 shows that the interactive effect between category and county-level towns was not significant. Therefore, it is valid to consider that the urban land accessibility are greatly affected by the educational facility category.

### 4.3. Analysis of the Urban Land Accessibility of the Locations of Educational Facilities in County-Level Towns across the Country

From the previous section, we can see that the comprehensive accessibility of educational facilities between the four typical towns was different but not significant. Therefore, the data of the four typical towns were combined and analyzed to obtain a more stable value. The comprehensive accessibility data were plotted on a box plot.

**Figure 6:**
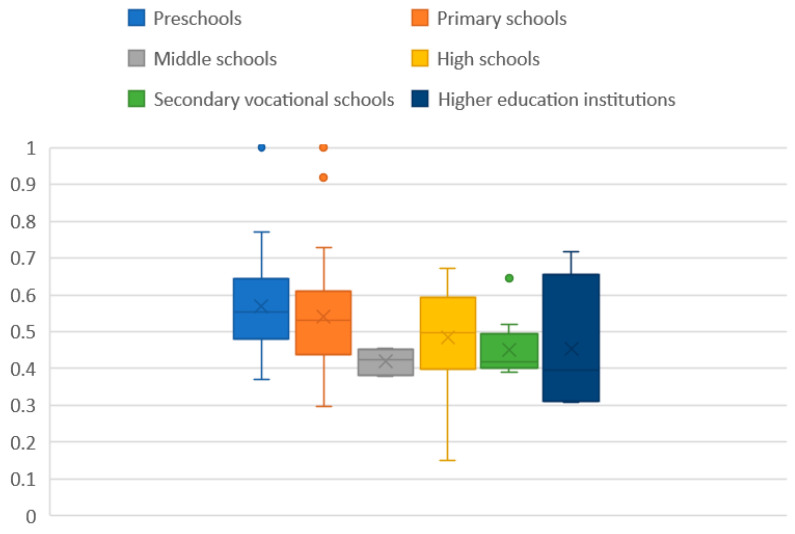
Box plot for comprehensive urban land accessibility of the locations of educational facilities at all levels in a typical county.

It can be seen from Figure 6 that there are four outliers. After the outliers were deleted, the data were imported into SPSS to determine the mean, standard deviation, maximum, minimum, and quantiles at 10, 20, 50, 60, and 90. The percentile calculation method of the urban land accessibility is as follows:

Sort the values of n variables from small to large; X(j) is the jth number in this sequence.

Calculate the exponent and set (n+1)P%=j+g, where *j* is the integer part and *g* is the decimal part.

(1) When g = 0: P percentile is *X*(*j*);

(2) When g≠0: P percentile is g∗X(j+1)+(1−g)∗X(j).

**Figure 7:**
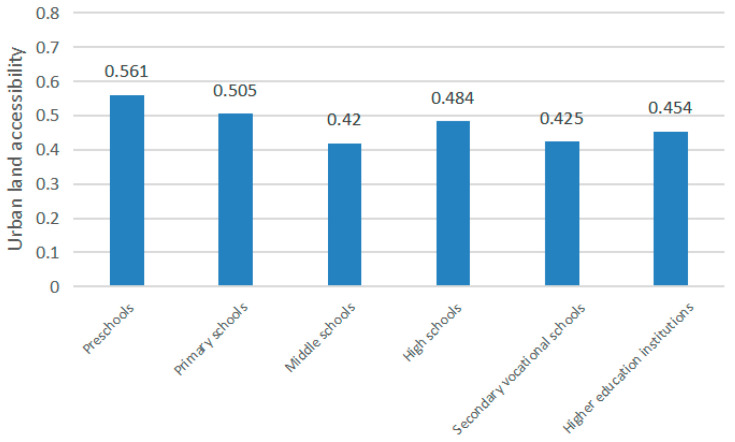
Graph of the average value of the comprehensive urban land accessibility of the locations of educational facilities at all levels.

It can be seen from Figure 7 that the average urban land accessibility of the locations of educational facilities at all levels is between 0.4 and 0.6, with slight differences.

The average values of the urban land accessibility of the locations of the educational facilities at all levels were sorted from high to low as kindergarten, elementary school, high school, colleges and universities, secondary vocational school, and junior high school. The reason for this phenomenon could be that most kindergarten and elementary school students need to be picked up by their parents every day and will therefore be in areas with higher accessibility. Starting with junior high school, there are more boarding students, and their parents do not have to drop them off and pick them up as much; thus, accessibility is slightly reduced.

According to the normality test in Section 4.2, the comprehensive urban land accessibility of the locations of educational facilities at all levels across the country can be considered to obey normal distribution. According to the 3σ rule of normal distribution, 68.27% of the data were in the range of (μ−σ, μ+σ) (Table 14).

## 5. Case Study of the Correlation between Medical and Health Facilities Categories and the Urban Land Accessibility

### 5.1. Analysis of the Characteristics of the Urban Land Accessibility of the Locations of Typical County Medical and Health Facilities

Based on the previously mentioned classification method, medical and health facilities were divided into three categories (i.e., hospitals, professional public health institutions, primary medical and health institutions) to study the urban land accessibility of the locations of medical facilities at all levels in typical counties. Like the previous section, this section studies the relationship between the levels of medical and health facilities and the urban land accessibility of their locations in Zhicheng Town, a water town in Changxing County; Zhao Town in Jintang County, Sichuan Province, in the southwest mountainous area; Qingcheng Town in Qingcheng County, Gansu Province, in the Loess Plateau; and Wu’an Town, in Wu’an City, Hebei Province, in the North China Plain.

**Figure 8:**
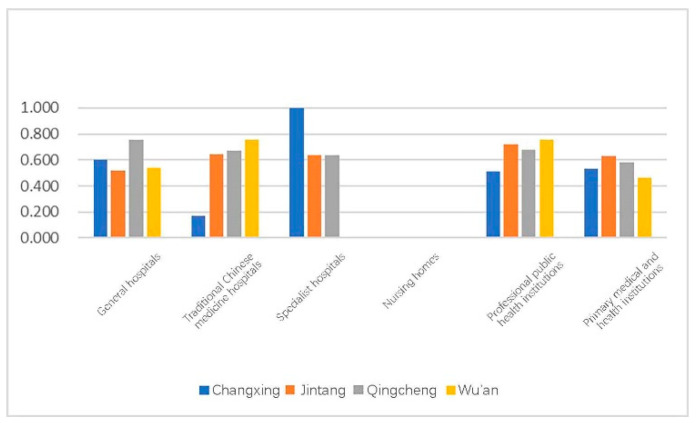
Graph of the average value of the comprehensive urban land accessibility of the locations of medical facilities at all levels in a typical county.

The averages of the four towns were compared (Figure 8).

Since there is only one traditional Chinese medicine hospital and one specialist hospital in Changxing County, the variance was too great to reliably determine statistical significance; thus, the data of these two categories are only for descriptive purposes. The data for other medical and health facilities at all levels fluctuated slightly between the different towns. Therefore, we could preliminarily determine that the average accessibility of educational facilities at all levels had nothing to do with the climate zone. The four counties were then combined and analyzed to obtain a more stable and accurate value.

### 5.2. Analysis of the Correlation between the Categories of Medical and Health Facilities and the Urban Land Accessibility in the County-Level Towns

Since there are few traditional Chinese medicine hospitals, specialist hospitals, and nursing homes in county-level towns, medical and health facilities were classified into three categories: hospitals, professional medical institutions, and primary medical and health institutions. This section mainly examines whether the categories of medical and health facilities, as well as their locations, have any effect on the urban land accessibility.

As in Section 4.2, first, normality and variance homogeneity tests were performed. According to the category classification, the *p* value of professional medical institutions is greater than 0.05, but the scatter points in the Q–Q plot are distributed on the diagonal. Thus, the high p value might have been caused by the relatively small sample size. Since the overall population obeys normal distribution, the distribution of professional medical institutions can be approximated as normal distribution here.

A p value of less than 0.05 indicates that the factor had a significant effect on the dependent variable. As shown in Table 15, towns have a significant effect on primary health facilities but have no significant effect on hospitals and professional medical facilities. This could be because the location of some community-oriented primary facilities is very arbitrary. By contrast, hospitals and professional medical facilities are carefully planned and considered, and their site selection is more scientific. Therefore, different towns have effects on the accessibility of primary medical facilities.

### 5.3. Analysis of the Locations of Medical and Health Facilities in County-Level Towns across the Country and the Urban Land Accessibility

Data for the four typical towns were combined and analyzed to create a box plot (Figure 8).

**Figure 9:**
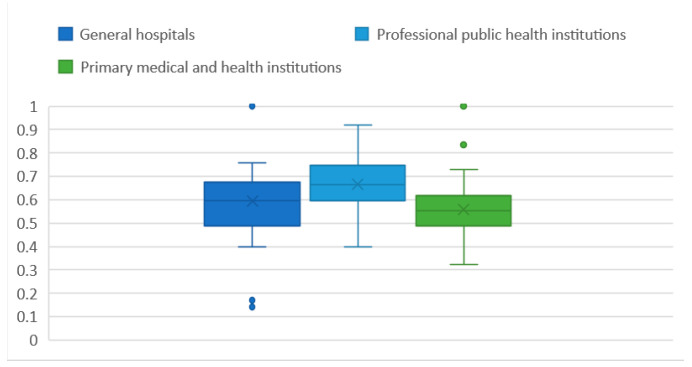
Box plot for the urban land comprehensive accessibility of the locations of medical facilities at all levels in a typical county.

Figure 9 shows there are five outliers. After the outliers were deleted, the data were imported into SPSS to analyze their mean, standard deviation, maximum, minimum, and percentiles at 20, 50, and 80.

The statistics in Table 16 indicate that the average accessibility of professional public health institutions is higher than that of primary medical and health institutions and general hospitals. The possible reason for this phenomenon is that general hospitals have a wider range of services and a larger scale. They are not suitable for locations with high accessibility. Hospitals do not require commuting the way schools do. There is no need to for locations with high accessibility. Most primary medical and health institutions in various communities are health service centers and health service stations. They have a relatively small service scope, target only nearby communities, and are more grassroots oriented; thus, their accessibility is low.

From the normality test in Section 4.2, we can assume that the distribution of medical and health facilities is normal. According to the 3σ rule of normal distribution, 68.27% of the data were in the range of (μ−σ, μ+σ) (Table 17).

## 6. Correlation Analysis on Carbon Emissions of Facility-Related Trip and Accessibility

In Section 4 and Section 5, it is testified that the category of public service facilities had a significant effect on the comprehensive urban land accessibility. Further, a 68% accessibility range of both education and medical care facilities was derived. This chapter analyzes the correlation between carbon emission of facility-related trip and urban land accessibility.

### 6.1. Calculation on Carbon Emissions of Facility-Related Trip

A micro carbon emission model is established by the trip distance and traffic mode of facility-related trips. Xu() summarized direct carbon emission coefficients of different traffic modes. These coefficients calibrated the carbon emission when one person travels one kilometre by a specific traffic mode. Thus, carbon emissions of single facility-related trips can be calculated as the multiplication of trip distance and the corresponding direct carbon emission coefficient.

Then, average carbon emissions of a single trip on public service facilities of different categories were calculated.

### 6.2. Correlation between Carbon Emissions of Trip on Education Facility and Accessibility

Average carbon emissions of a single trip on education facilities were calculated and classified by categories as kindergartens, primary schools, middle schools, high schools and others (mainly include secondary vocational schools and higher education institutions). Comprehensive accessibility and single trip carbon emissions were plotted as a scatter diagram, and the trend line was drawn by least square method.

**Figure 10:**
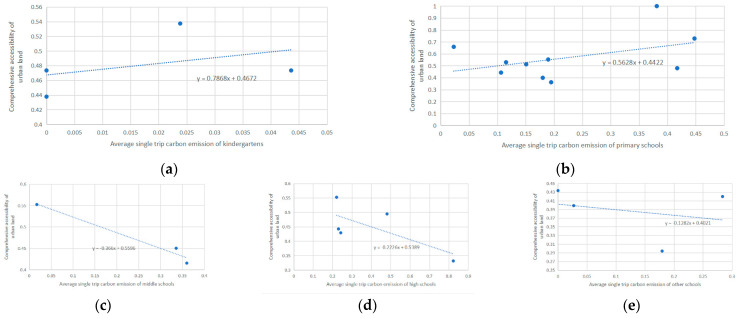
(**a**) Correlation between average single trip carbon emissions of kindergartens and comprehensive accessibility, (**b**) Correlation between average single trip carbon emissions of primary schools and comprehensive accessibility, (**c**) Correlation between average single trip carbon emissions of middle schools and comprehensive accessibility, (**d**) Correlation between average single trip carbon emissions of high schools and comprehensive accessibility, (**e**) Correlation between average single trip carbon emissions of other schools and comprehensive accessibility.

As shown in Figure 10, single trip carbon emissions of kindergartens and primary schools has a positive correlation with accessibility of their locations. Theoretically, facilities with higher accessibility have better locations and are more accessible. However, they caused more carbon emissions. Likewise, facilities with low accessibility should be remote and hard to access. Inversely, they caused less carbon emission. This is because kindergartens and primary schools usually have relatively small service scopes. Parents care more about convenience when selecting schools for children. These facilities are less accessible for the whole county but more accessible for the districts they serve. Education facilities with low accessibility usually focus on several districts around them, which makes it easier for students to access and causes less carbon emissions. While education facilities with high accessibility have large service scopes and serve more districts. Even students who live far away could choose these schools, which causes higher single trip carbon emissions.

When it comes to middle schools, high schools and other education facilities, parents care more about teaching quality than their locations. Many middle school students and high school students live at schools, which lowers commuting frequency. Therefore, education facilities with higher accessibility have low single trip carbon emissions.

### 6.3. Correlation between Carbon Emissions of Trip on Medical Care Facility and Accessibility

Average carbon emissions of a single trip on medical care facilities were calculated and classified by categories as hospitals and primary medical and health institutions. Comprehensive accessibility and single trip carbon emissions were plotted as scatter diagram. And the trend line was drawn by least square method.

**Figure 11:**
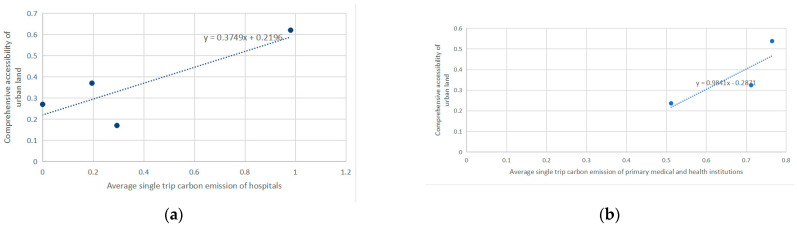
(**a**) Correlation between average single trip carbon emissions of hospitals and comprehensive accessibility, (**b**) Correlation between average single trip carbon emissions of primary medical and health institutions and comprehensive accessibility.

As shown in Figure 11, single trip carbon emissions of hospitals and primary medical and health institutions has a positive correlation with accessibility of their locations. The reason could be medical care facilities with low accessibility are located in less developed areas. People are more likely to choose green and low-carbon traffic modes to get there in their relatively small service scope. Meanwhile, medical care facilities with high accessibility are usually high standard hospitals, which are shared by several towns. There are cases in trip data where residents travelled across towns to get medical care service and travelled by car due to urgent situations. These cases contributed high carbon emissions to the overall trip emissions.

### 6.4. Location Choice of Public Service Facilities Considering Accessibility and Carbon Emissions

Synthesizing former analysis on different categories of public service facilities and linear regression models, the corresponding relation between carbon emissions level and urban land accessibility was established. In Table 18 and Table 19, comprehensive accessibility was restricted in 68% range (Table 14 and Table 17) of overall accessibility to make sure no irrational decision would be made in extreme purse of low carbon emissions.

Furthermore, an innovative planning process of public service facilities was proposed considering existing practices:

(1) Divide the administrative region of a city into groups and decide the amount of public service facilities according to population and area.
(2) Divide the planning scope into 400 m × 400 m grids as a basic unit.
(3) Calculate the comprehensive accessibility of each location.
(4) Choose a suitable range of comprehensive accessibility in the corresponding relation table according to carbon emissions expectation.
(5) Choose suitable locations according to the range of comprehensive accessibility.
(6) Choose one of those locations considering actual conditions.

## 7. Conclusions

This study focuses on the two most extensive and basic categories of public service facilities in county-level towns, which are educational and medical facilities. This study has built connections between transportation conditions with locations of public service facilities. This connection was interpreted as the correlation between urban land accessibility with locations of public service facilities. The purpose of the current study is to find out the distribution of the accessibility of the urban land grid where different types of public service facilities are located. Thus, innovative and quantitative indicators can be defined to reform the location selection during planning of public service facilities. These findings suggest that in general, the urban land accessibility of the locations of public service facilities follows a normal distribution. Different categories of facilities show various patterns on their locations’ urban land accessibility. This research lends support to including innovative and quantitative indicators into planning of public service facilities, which considers transportation conditions. It optimizes traditional methods by relying on not only macroscopic indicators. One of the applications of urban land accessibility could be reducing carbon emissions caused by trips related to facilities, responding to the call of the United Nations at the 2020 Climate Ambition Summit. We readily acknowledge that there are problems with the limitation of the number of samples. More data from counties in different stages of development could make the results more convincing. Future research may focus on more applications of urban land accessibility on planning of public service facilities. One promising approach is to study on how we can improve accessibility by constructing transportation infrastructure to meet the needs of certain categories of public service facilities.

## Figures and Tables

**Table 1:** Facility categories in the Standard for Urban Public Service Facilities Planning.

School Category	Specific Category
Primary and secondary schools	Primary schools
	Middle schools
	General high schools
	Nine-year schools
	Combined middle and high schools
Special Education Schools	Schools for the blind or the deaf, schools for students with intellectual disabilities, and multidisciplinary special education schools
Secondary vocational schools	Vocational high schools
	Secondary professional schools
	Technical schools
Higher education institutions	Universities
	Colleges
	Higher vocational school
	Higher professional schools
	Party schools of the Communist Party of China

**Table 2:** Educational facility categories in the Standard for Urban Public Service Facilities Planning.

Facility Category	Facility Type	
Hospitals	General hospitals	
	Traditional Chinese medicine hospitals	
	Specialist hospitals	Psychiatric hospitals
		Infectious disease hospitals
		Children’s hospital
		Other specialty hospitals
	Nursing homes	
Professional public health institutions	Emergency centers (stations)	
	Blood collection and supply institutions	
	Maternal and child health hospitals	
	Centers for disease control and prevention	
Primary medical and health institutions	Community health service center	
	Community health service stations	

**Table 3:** Characteristic values of urban land accessibility of locations of various levels of educational facilities in Zhicheng Town, Changxing City.

School Category	Specific Category	Max	Min	Mean
Preschool	Preschools	0.371	1.000	0.546
Primary and secondary schools	Primary schools	0.298	1.000	0.558
	Middle schools	0.450	0.450	0.450
	High schools	0.298	0.552	0.429
Secondary vocational schools	Vocational high schools, secondary professional schools, technical schools	0.389	0.521	0.430
Higher education institutions	Universities, colleges, higher professional schools, party schools the Communist Party of China	0.307	0.469	0.366

**Table 4:** Normality test of the comprehensive urban land accessibility of the sample population.

	Kolmogorov-Smirnov ^a^			Shapiro-Wilk		
	Statistic	df	Sig.	Statistic	df	Sig.
Comprehensive accessibility	0.051	121	0.200 *	0.987	121	0.290

^a^ Lilliefors significant level correction. * This is the lower limit of the true significant level.

**Table 5:** Normality test table based on the towns where facilities are located.

Statistic	Town	Kolmogorov-Smirnov ^a^			Shapiro-Wilk		
		Statistic	df	Sig.	Statistic	df	Sig.
Comprehensive accessibility	Changxing	0.107	45	0.200 *	0.947	45	0.038
	Jintang	0.122	35	0.200 *	0.957	35	0.191
	Qingcheng	0.152	20	0.200 *	0.947	20	0.318
	Wu’an	0.157	24	0.131	0.939	24	0.151

^a^ Lilliefors significant level correction * This is the lower limit of the true significant level.

**Table 6:** Normality test based on the category of educational facilities.

Statistic	Category	Kolmogorov-Smirnov ^a^			Shapiro-Wilk		
		Statistic	df	Sig.	Statistic	df	Sig.
Comprehensive accessibility	1	0.122	35	0.200 *	0.957	35	0.191
	2	0.152	20	0.200 *	0.947	20	0.318
	3	0.157	24	0.131	0.939	24	0.151
	4	0.158	47	0.005	0.853	47	0.000

^a^ Lilliefors significant level correction * This is the upper limit of the true significant level.

**Table 7:** Comparison of standard deviations in different county-level towns.

County	Mean	N	Standard Deviation
Changxing	0.469	45	0.110
Jintang	0.554	35	0.095
Qingcheng	0.580	20	0.098
Wu’an	0.471	24	0.190
Total	0.511	124	0.131

**Table 8:** Comparison of standard deviations of comprehensive accessibility of different categories.

Category	Mean	N	Standard Deviation
1	0.569	50	0.121
2	0.517	37	0.163
3	0.470	11	0.113
4	0.449	16	0.151
5	0.452	8	0.087
6	0.453	4	0.190
Total	0.519	126	0.144

**Table 9:** Variance analysis of comprehensive accessibility of educational facilities by category.

Comprehensive Urban Land Accessibility					
	Sum of Squares	df	Mean Square	F	Significance
Between groups	0.285	5	0.057	2.947	0.015
Within groups	2.323	120	0.019		
Total	2.609	125			

**Table 10:** One-way analysis of variance results table for preschools.

Source	Type III Sum of Squares	df	Mean Square	F	Sig.
Calibration model	0.056 ^a^	3	0.019	1.280	0.292
Intercept	12.288	1	12.288	844.628	0.000
Town	0.056	3	0.019	1.280	0.292
Error	0.669	46	0.015		
Total	16.957	50			
Corrected total	0.725	49			

^a^ R^2^ = 0.077 (adjusted R^2^ = −0.017).

**Table 11:** One-way analysis of variance results table for primary schools.

Source	Type III Sum of Squares	df	Mean Square	F	Sig.
Calibration model	0.007 ^a^	3	0.002	0.085	0.968
Intercept	8.520	1	8.520	294.308	0.000
Town	0.007	3	0.002	0.085	0.968
Error	0.955	33	0.029		
Total	10.867	37			
Corrected total	0.963	36			

^a^ R^2^ = 0.008 (adjusted R^2^ = −0.083).

**Table 12:** One-way analysis of variance results table for middle and high schools.

Source	Type III Sum of Squares	df	Mean Square	F	Sig.
Calibration model	0.123 ^a^	3	0.041	2.661	0.072
Intercept	5.477	1	5.477	356.437	0.000
County-level town	0.123	3	0.041	2.661	0.072
Error	0.353	23	0.015		
Total	6.140	27			
Corrected total	0.476	26			

^a^ R^2^ = 0.258 (adjusted R^2^ = 0.161).

**Table 13:** Variance analysis of the interaction between categories and towns on the comprehensive urban land accessibility.

Dependent Variable: Comprehensive Urban Land Accessibility					
Source	Type III Sum of Squares	df	Mean Square	F	Sig.
Calibration model	0.684 ^a^	19	0.036	1.982	0.015
Intercept	13.434	1	13.434	739.800	0.000
Category	0.139	5	0.028	1.531	0.186
Town	0.144	3	0.048	2.641	0.053
Category × Town	0.299	11	0.027	1.496	0.144
Error	1.925	106	0.018		
Total	36.585	126			
Corrected total	2.609	125			

^a^ R^2^ = 0.262 (adjusted R^2^ = 0.130).

**Table 14:** 68% range of comprehensive urban land accessibility of the locations of various levels of educational facilities nationwide.

Facility Level	68% Accessibility Range
Preschools	0.455–0.667
Primary schools	0.401–0.609
Middle schools	0.382–0.458
High schools	0.365–0.603
Special education schools	0.536
Secondary vocational schools	0.381–0.469
Higher education institutions	0.264–0.644

**Table 15:** Significance level of analysis of variance.

Analysis of Variance	Significance Level
The impact of facility category on comprehensive accessibility	0.043
The impact of town on the comprehensive accessibility of primary medical facilities	0.044
The impact of town on the comprehensive accessibility of hospitals	0.727
The impact of town on the comprehensive accessibility of professional healthcare facilities	0.375
The impact of the interaction between facility category and town on the comprehensive accessibility of hospitals	0.114

**Table 16:** Characteristic values of medical and health facilities at all levels across the country.

Statistic		General Hospitals	Professional Public Health Institutions	Primary Medical and Health Institutions
N	Valid	21	9	90
	Missing	69	81	0
Mean		0.5785518	0.666777	0.5522949
Standard deviation		0.09650820	0.1425749	0.09413388
Min		0.39793	0.3992	0.32368
Max		0.75809	0.9184	0.72839
Percentile	20	0.4901900	0.593630	0.4743845
	50	0.5952300	0.663300	0.5528312
	80	0.6585620	0.771300	0.6439653

**Table 17:** 68% accessibility range of medical and health facilities nationwide.

Facility Level	68% Accessibility Range
General hospitals	0.482–0.675
Professional public health institutions	0.524–0.809
Primary medical and health institutions	0.458–0.646

**Table 18:** Corresponding relation of comprehensive accessibility and education facilities.

Category of Facility	Carbon Emission	Comprehensive Accessibility
Kindergartens	0–0.05	0.467–0.507
	0.05–0.1	0.507–0.526
	0.1–0.15	0.546–0.585
	0.15–0.20	0.585–0.625
Primary schools	0–0.05	0.442–0.470
	0.05–0.1	0.470–0.498
	0.1–0.15	0.498–0.527
	0.15–0.20	0.527–0.555
	0.20–0.25	0.555–0.583
Middle schools	0.25–0.30	0.450–0.468
	0.30–0.35	0.432–0.450
	0.35–0.40	0.413–0.432
	0.40–0.45	0.395–0.413
	0.45–0.50	0.376–0.395
High schools	0–0.10	0.517–0.539
	0.10–0.20	0.494–0.517
	0.20–0.30	0.472–0.494
	0.30–0.40	0.450–0.472
	0.40–0.50	0.428–0.450
	0.50–0.60	0.405–0.428

**Table 19:** Corresponding relation of comprehensive accessibility and medical care facilities.

Category of Facility	Carbon Emission	Comprehensive Accessibility
Hospitals	0.4–0.6	0.370–0.445
	0.6–0.8	0.445–0.520
	0.8–1.0	0.520–0.595
Primary medical care and health institutions	0.4–0.6	0.107–0.303
	0.6–0.8	0.303–0.500
	0.8–1.0	0.500–0.697

## Data Availability

The data presented in this study are available on request from the corresponding author. The data are not publicly available due to the requirement of our funder.

## References

[1] Urbanization Level Significantly Improved, Cities Exhibiting a New Look—Report 11 on the Economic and Social Development Achievements in the 40 Years of Reform and Opening Up. http://www.stats.gov.cn/ztjc/ztfx/ggkf40n/201809/t20180910_1621837.html.

[2] Toregas C., Swain R., ReVelle C. (1971). The location of emergency service facilities. Oper. Res..

[3] Church R., ReVelle C. (1974). The maximal covering location problem. Pap. Reg. Sci. Assoc..

[4] Hakimi S.L. (1964). Optimum locations of switching centers and the absolute centers and medians of a graph. Oper. Res..

[5] Hakimi S.L. (1986). p-median theorems for competitive location. Ann. Oper. Res..

[6] Zheng Z., Morimoto T., Murayama Y. (2020). Optimal Location Analysis of Delivery Parcel-Pickup Points Using AHP and Network Huff Model: A Case Study of Shiweitang Sub-District in Guangzhou City, China. ISPRS Int. J. Geo. Inf..

[7] Song Z., Yan T., Ge Y. (2018). Spatial Equilibrium Allocation of Urban Large Public General Hospitals Based on the Welfare Maximization Principle: A Case Study of Nanjing, China. Sustainability.

[8] Cheng M., Cui X. (2020). Spatial Optimization of Residential Care Facility Configuration Based on the Integration of Modified Immune Algorithm and GIS: A Case Study of Jing’an District in Shanghai, China. Int. J. Environ. Res. Public Health.

[9] Ni J., Qian T., Xi C., Rui Y., Wang J. (2016). Spatial Distribution Characteristics of Healthcare Facilities in Nanjing: Network Point Pattern Analysis and Correlation Analysis. Int. J. Environ. Res. Public Health.

[10] Shirowzhan S., Tan W., Sepasgozar S.M.E. (2020). Digital Twin and CyberGIS for Improving Connectivity and Measuring the Impact of Infrastructure Construction Planning in Smart Cities. ISPRS Int. J. Geo. Inf..

[11] Peungnumsai A., Miyazaki H., Witayangkurn A., Kim S.M. (2020). A Grid-Based Spatial Analysis for Detecting Supply–Demand Gaps of Public Transports: A Case Study of the Bangkok Metropolitan Region. Sustainability.

[12] Kong X., Liu Y., Wang Y., Tong D., Zhang J. (2017). Investigating Public Facility Characteristics from a Spatial Interaction Perspective: A Case Study of Beijing Hospitals Using Taxi Data. ISPRS Int. J. Geo. Inf..

[13] Talen E. (1998). Visualizing fairness: Equity maps for planners. J. Am. Plan. Assoc..

[14] Luo W., Wang F. (2003). Measures of spatial accessibility to health care in a GIS environment: Synthesis and a case study in the Chicago region. Environ. Plan. B.

[15] Boone C.G., Buckley G.L., Grove J.M., Sister C. (2009). Parks and people: An environmental justice inquiry in Baltimore, Maryland. Ann. Assoc. Am. Geogr..

[16] Weber J., Kwan M.P. (2002). Bringing time back in: A study on the influence of travel time variations and facility opening hours on individual accessibility. Prof. Geogr..

[17] Neutens T., Delafontaine M., Scott D.M., De Maeyer P. (2012). A GIS-based method to identify spatiotemporal gaps in public service delivery. Appl. Geogr..

[18] Ahmed S., Adams A.M., Islam R., Hasan S.M., Panciera R. (2019). Impact of traffic variability on geographic accessibility to 24/7 emergency healthcare for the urban poor: A GIS study in Dhaka, Bangladesh. PLoS ONE.

[19] Hu X., Yang K., Zhang C. (2020). Optimization of Preparation Conditions for Side-Emitting Polymer Optical Fibers Using Response Surface Methodology. Polymers.

[20] Gao W., Tu R., Li H., Fang Y., Que Q. (2020). In the Subtropical Monsoon Climate High-Density City, What Features of the Neighborhood Environment Matter Most for Public Health?. Int. J. Environ. Res. Public Health.

